# Socio-Economic Inequalities in the Use of Postnatal Care in India

**DOI:** 10.1371/journal.pone.0037037

**Published:** 2012-05-18

**Authors:** Abhishek Singh, Sabu S. Padmadas, Udaya S. Mishra, Saseendran Pallikadavath, Fiifi A. Johnson, Zoe Matthews

**Affiliations:** 1 Department of Public Health and Mortality Studies, International Institute for Population Sciences, Mumbai, India; 2 Centre for Global Health, Population, Poverty and Policy and Division of Social Statistics and Demography, University of Southampton, Southampton, United Kingdom; 3 Center for Development Studies, Thiruvanathpuram, India; 4 Global Health and Social Care Unit, School of Health Sciences and Social Work, University of Portsmouth, Portsmouth, United Kingdom; Kenya Medical Research Institute - Wellcome Trust Research Programme, Kenya

## Abstract

**Objectives:**

First, our objective was to estimate socio-economic inequalities in the use of postnatal care (PNC) compared with those in the use of care at birth and antenatal care. Second, we wanted to compare inequalities in the use of PNC between facility births and home births and to determine inequalities in the use of PNC among mothers with high-risk births.

**Methods and Findings:**

Rich–poor ratios and concentration indices for maternity care were estimated using the third round of the District Level Household Survey conducted in India in 2007–08. Binary logistic regression models were used to examine the socio-economic inequalities associated with use of PNC after adjusting for relevant socio-economic and demographic characteristics. PNC for both mothers and newborns was substantially lower than the care received during pregnancy and child birth. Only 44% of mothers in India at the time of survey received any care within 48 hours after birth. Likewise, only 45% of newborns received check-up within 24 hours of birth. Mothers who had home births were significantly less likely to have received PNC than those who had facility births, with significant differences across the socio-economic strata. Moreover, the rich-poor gap in PNC use was significantly wider for mothers with birth complications.

**Conclusions:**

PNC use has been unacceptably low in India given the risks of mortality for mothers and babies shortly after birth. However, there is evidence to suggest that effective use of pregnancy and childbirth care in health facilities led to better PNC. There are also significant socio-economic inequalities in access to PNC even for those accessing facility-based care. The coverage of essential PNC is inadequate, especially for mothers from economically disadvantaged households. The findings suggest the need for strengthening PNC services to keep pace with advances in coverage for care at birth and prenatal services in India through targeted policy interventions.

## Introduction

The highest risk of death for both the mother and her newborn occurs at the time of childbirth or immediately in the period after birth. Ensuring appropriate postnatal care is critical to safeguarding maternal and newborn health [1]–[3]. More than two-thirds of newborn deaths occur within the first week after birth and of these, most deaths occur in the first 24 hours of birth [4]. This is also the case with maternal deaths where almost two-thirds tend to occur in the postnatal period [5]. India is no exception to this. About 39% of neonatal deaths occur on the first day of life in India, about 57% during the first three days [6] and the majority of maternal deaths occur between the third trimester and the end of the first week after birth [7].

Promoting antenatal care and skilled attendance at birth is clearly not enough for improving maternal and child health. The WHO guidelines on postnatal care recommend essential routine postnatal care for all mothers and their newborns, extra care for low birth weight and small babies, and early identification and referral or management of emergency conditions. The guidelines further recommend postnatal visits within 6 to 12 hours after birth, and follow-up visits from 3 to 6 days, at 6 weeks, and then at 6 months [8]. Strategies aimed at promoting universal access to postnatal care have been recommended for several years [9] and these interventions can have measurable and sustained impact in reducing neonatal and maternal mortality [1]. Yet, despite governmental initiatives and policy efforts, there is a lack of follow-up after childbirth. Moreover, mothers often only seek postnatal care in the event of complications after birth. Poverty, lack of schooling, poor knowledge, and inadequate follow-up services in healthcare systems deter women from seeking postnatal care. To date, there has not been any systematic national level analysis of population data to understand the levels and socio-economic differentials in the use of postnatal care in India. Little evidence exists on the extent and timing of postnatal care and how these differ between women who received home and institutional care at birth.

A continuum of care throughout pregnancy and the postpartum period is critically important in India where both mothers and children are vulnerable to a range of health risks resulting from the vicious cycle of malnutrition and poverty. Moreover, despite a recent decline in infant mortality rates, there is little subsequent improvement in most Indian states [10]–[11]. The infant mortality rate was around 57 per 1000 live births in 2005–06 and ranged between as low as 15 in Kerala to as high as 73 in Uttar Pradesh [11]. Maternal mortality rates were also high at 212 deaths per 100,000 live births, which varied widely across Indian states [12]. The Government of India introduced several policy measures and interventions to tackle the burden of infant and maternal mortality by reorienting the National Population Policy (2000), the National Health Policy (2002), the Reproductive and Child Health Programme (Phase I – 1997–2004, Phase II – 2005–2010), and the National Rural Health Mission (2005–2012) – wherein improving postnatal coverage was envisaged as one of the key intervention strategies to reduce infant and maternal mortality rates [13]. Unfortunately, although the policies have rightly emphasised on components such as skilled attendance and antenatal and institutional care at birth, they have overlooked the need to strengthen postnatal care within the reproductive health services.

**Table 1:** Use of antenatal, delivery and postnatal care services for the last pregnancy recorded in the five years preceding the survey, India, 2007–08.

Type of services received	%	Number of mothers
**Mother received any antenatal care**YesNo	73.0526.95	158,40458,427
**Mother received 4 or more ANC visits**Less than four visitsFour or more visits	57.4942.51	89,47566,172
**During antental care, mother received any advice on institutional delivery**YesNo	46.0253.98	72,85685,470
**Mother experienced any complication during delivery**YesNo	58.5641.44	133,60094,544
**Place of childbirth**HomeInstitution	56.8243.18	123,20893,628
**Mode of delivery**NormalC-section	91.948.06	199,33017,478
**Mother received any postnatal check-up within 48 hours of delivery**YesNo	44.0655.94	95,536121,300
**Newborn received any postnatal check-up within 24 hours of birth**YesNo	44.9955.01	95,837117,161
**Number of postnatal check-ups in first 10 days after birth**Less than two check-upsTwo or more check-ups	38.4261.58	37,04759,369
**Place of first postnatal check-up**Government facilityPrivate facility	44.8055.20	43,23053,275

Note: Frequencies in some variables may not add to 219, 388 due to missing cases.

In general, postnatal care uptake has been limited in south Asia [1], [14]–[16] and particularly in India. According to the 2005–06 National Family Health Survey (NFHS-3), only 42% of women reported receiving postnatal check-up after their recent birth [11]. Of these, only about a third received check-up within the first two days after birth. Unfortunately, the NFHS-3 collected data on postnatal care from only mothers who had given birth in a health facility. Postnatal care is under-researched in India at the national level, except a study conducted in the state of Madhya Pradesh [17], one conducted in southern India [18] and another rural-based study [19]. However, none of these studies explicitly addressed the socio-economic inequalities in postnatal care use.

This paper quantifies the extent of socio-economic inequalities in the use of various components of postnatal care (PNC) in India. First, the study examines inequalities in use of PNC compared with those in utilization of care at birth and antenatal care. Second, given new large-sample survey data collected in 2007–08 which asked specific questions on PNC we were able to compare inequalities in the use of PNC between facility births and home births. Additionally, we estimated socio-economic inequalities in the case of births that were at risk e.g. cesarean births and births with maternal complications.

**Table 2:** Rich-Poor ratio and concentration index for use of postnatal and other services, India, 2007–08.

**Type of services received**	**% among total**			
	**Richest**	**Poorest**	**Ratio**	**Concentration Index**
	**(1)**	**(2)**	**(1/2)**	
Mother received any ANC	93.6	54.8	1.7***	0.047***
Mother received 4 or more ANC visits	67.7	19.7	3.4***	0.070***
During ANC, received advice on institutional delivery	60.1	32.1	1.9***	0.126***
Institutional birth	79.9	19.1	4.2***	0.087***
Mother received any PNC check-up within 48 hours of birth	77.1	22.7	3.4***	0.078***
Newborn received PNC check-up within 24 hours of birth	78.6	23.3	3.4***	0.243***
Baby received two or more PNC check-ups within first 10 days of life	75.4	43.00	1.8***	0.104***
Baby was checked at				
(a) Government facility	34.8	47.4	0.7***	−0.079***
(b) Private facility	65.2	52.6	1.2***	0.301***

Note: ***P<0.001, Differences were tested for significance using one way ANOVA. Associations between the dependent and independent variables were tested using chi-square test. All the associations were significant at p<0.001 level.

## Methods

### Ethics Statement

The study is based on a publically available secondary data set with no identifiable information on the survey participants. This dataset is available in the public domain for research use and hence no formal approval from the institutional review board is required. So, no ethics statement required for this work.

### Data

We used data from the third round of the District Level Household Survey (DLHS-3) conducted in 2007–08 in 601 districts from 34 states and union territories of India. DLHS-3 adopted a multi-stage stratified systematic sampling design which resulted in national and state-representative samples after applying sampling weights to control for complex survey design [7]. A total of 643,944 ever married women aged 15–49 years and 166,260 unmarried women aged 15–24 years were interviewed in the survey. Data on postnatal care were collected from all women who had a birth in the five years preceding the survey date, irrespective of whether they sought delivery care at home or in a health facility.

### Outcome Variables

The outcome variable considered in the analysis was the type and timing of postnatal care. The availability of detailed information in DLHS-3 provided us with opportunities to analyze inequalities in type and timing of postnatal care by place of birth, complications during birth, and cesarean birth. In addition, the analysis considered antenatal care and care at birth (home or facility).

### Exposure Variables

The main explanatory variable of interest was a proxy measure of household economic status. Direct data on income or expenditure are not available in the DLHS-3 and in circumstances where such data available in retrospective surveys are subject to reporting bias, a wealth index is computed based on the ownership of household assets and consumer durables [20]–[26]. Other demographic and social variables that influence maternal health care behaviour were also added including woman’s level of education, her place and region of residence, birth order, and gender status of the child.

**Figure 1:**
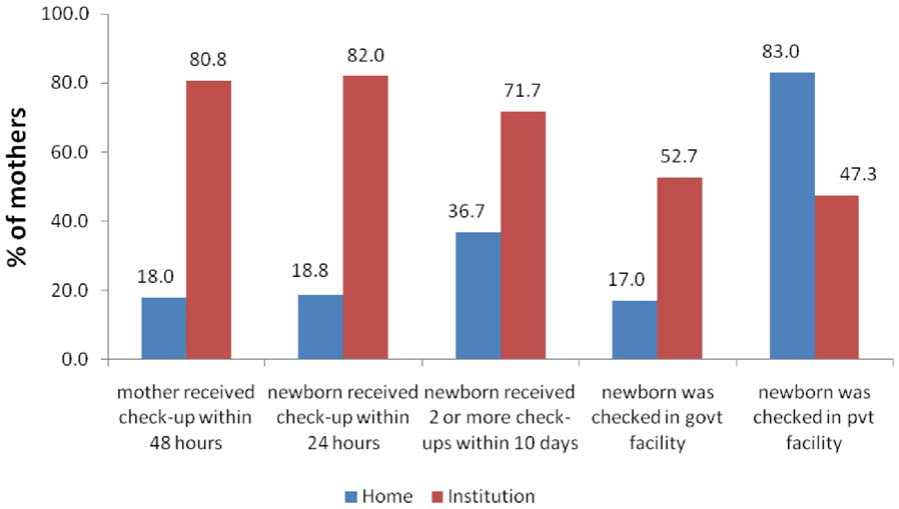
Differentials in postnatal care by place of childbirth.

**Table 3:** Concentration index for use of postnatal care by place of delivery, India, 2007–08.

Services	Concentration index	
	Home births	Facility births
Mother received any PNC check-up within 48 hours of birth	0.027***	0.027***
Newborn received PNC check-up within 24 hours of birth	0.182***	0.054***
Baby received two or more PNC check-ups within first 10 days of life	0.073***	0.061***
Baby was checked at		
(a) Government facility	0.015***	−0.166***
(b) Private facility	0.157***	0.255***

Note: ***P<0.001.

### Methods

Two measures were used to assess the extent of inequalities in the use of PNC: the rich-poor ratio (computed by dividing the top quintile by the bottom quintile of the wealth index) and the concentration index (CI - which measures the relationship between accumulated proportions of mothers ranked by their socio-economic status against the cumulative proportion of postnatal care use) [25], [27]–[29]. The values of the CI range from −1 to 1. A value equal to 1 or −1 indicates that only the richest or the poorest mothers use postnatal care respectively. These measures were also used to compare the differences in PNC with those receiving antenatal care and care at birth between home and facility and between births with and without maternal complications. Binary logistic regression models were fitted to assess the adjusted effect of wealth quintile on the likelihood of using PNC after adjusting for antenatal and childbirth care, and other relevant socio-economic and demographic characteristics. The explanatory variables were initially screened for potential multicollinearity before considering those in the regression models.

**Table 4:** Adjusted probabilities of postnatal care use by place of delivery, India, 2007–08.

Covariate & Category	Home delivery					Institutional delivery				
	Mother received PNC check-up within48 hours of birth^1^	Newborn receivedPNC check-up within24 hoursof birth^1^	Baby received2 or more PNCcheck-ups within10 days^2^	Baby received PNC check-up in government facility^1^	Baby received PNC check-up in private facility^1^	Mother received PNC check-upWithin48 hoursof birth^1^	Baby received PNCcheck-upwithin24 hoursof birth^1^	Baby received2 or morePNCcheck-ups within10 days^1^	Baby received PNC check-up in government facility^1^	Baby received PNC check-up in private facility^1^
**Wealth index**										
Poorest	0.11	0.12	0.27	0.09	0.09	0.60	0.60	0.47	0.75	0.12
Poorer	0.14	0.15	0.31	0.09	0.11	0.66	0.66	0.50	0.71	0.16
Middle	0.18	0.19	0.38	0.11	0.14	0.72	0.73	0.59	0.68	0.20
Richer	0.26	0.27	0.42	0.12	0.20	0.79	0.80	0.67	0.58	0.29
Richest	0.35	0.38	0.53	0.11	0.30	0.88	0.89	0.77	0.37	0.52
***Richest-Poorest***	0.24	0.26	0.26	0.02	0.21	0.28	0.29	0.30	−0.38	0.40

Note: The means were adjusted for mother’s education, place of residence, region of residence, health insurance coverage, birth order, mother received any ANC, and sex of the child. 1 - Wald test significant at p<0.001, 2 - Wald test significant at p<0.05.

## Results

### Inequalities in Use of Services–comparing PNC Use with ANC and Care at Birth

Only 44% of the mothers interviewed in the survey received any PNC check-up within 48 hours of giving birth (Table 1). Moreover, only 45% of the newborns were checked within 24 hours. Around 62% of the babies did, however eventually receive two or more check-ups within the first 10 days after birth. As expected, a majority of these babies were examined in a private facility (55%).

**Figure 2:**
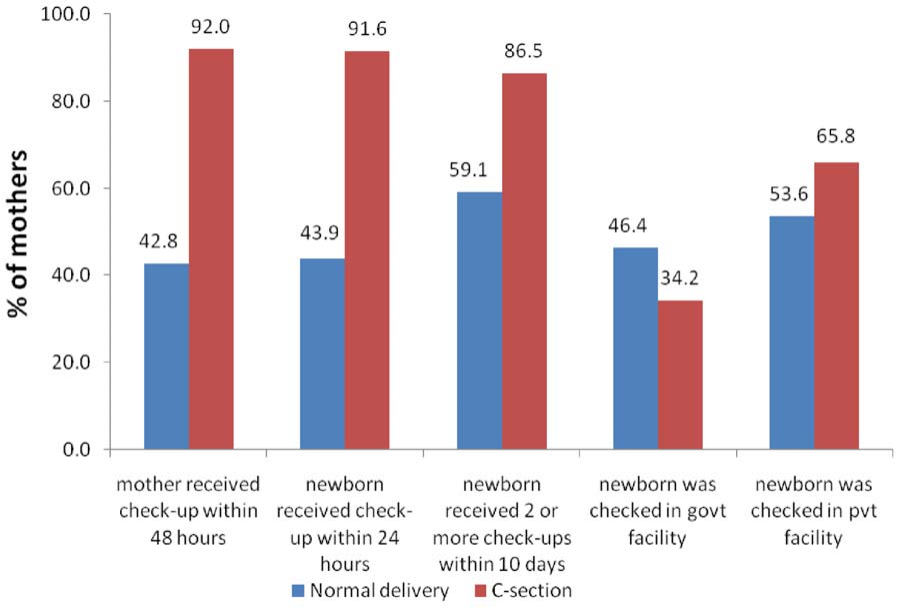
Differentials in postnatal care by mode of childbirth.

In contrast antenatal care was utilised by more women – about 73% of the mothers reported availing some form of care for their recent birth. However, only about 42% received the recommended four or more visits. Only 46% of women received advice on institutional care for childbirth. About 57% of the births took place at home or at places other than a health facility and about 58% of the mothers reported experiencing complications during childbirth. The majority of women had vaginal births while about 8% were cesarean sections.

Rich-poor ratios are presented in Table 2. Findings suggest enormous socio-economic inequalities in use of PNC services. Moreover, the socio-economic inequalities also varied across the continuum of care consisting of antenatal, birth and postnatal care services respectively. For instance, the rich-poor ratio varied from as low as 1.7 in case of receipt of any form of ANC to as high as 3.4 as regard to the compliance with 4 or more ANC visits. The rich were also four times more likely to have had an institutional birth when compared to the poor. The use of PNC was particularly unequal with the rich utilizing these services three times more compared with the poor. The rich-poor ratio in case of babies receiving two or more check-ups within the first 10 days after birth, however, was only 1.8. Further, babies belonging to the richer households were more likely to be examined in a private facility compared to the poor who were more likely to be examined in the government facilities.

Estimated concentration indices further confirm the wide socio-economic inequalities in the use of selected services (Table 2). The highest inequality was found in the use of PNC for newborns within 24 hours of birth. The rich were much more likely than the poor to get their babies seen by a health worker within 24 hours of birth. In addition, they were also more likely than the poor to get their newborns examined in a private facility. On the other hand, poor were more likely to receive a newborn check-up in a government facility. The rich were also relatively better in terms of receiving ANC advice than the poor. Findings clearly suggest that PNC was particularly unequal compared to other services.

**Table 5:** Concentration indices for use of postnatal care by maternal complications, India, 2007–08.

Services	*Caesarean births*	*Births with maternal complications*
Mother received any PNC check-up within 48 hours of birth	0.015***	0.074***
Newborn received PNC check-up within 24 hours of birth	0.034***	0.236***
Baby received two or more PNC check-ups within first 10 days of life	0.018***	0.109***
Baby was checked at		
(a) Government facility	−0.176***	−0.060***
(b) Private facility	0.240***	0.288***

Note: ***P<0.001.

### Inequality in Utilization of PNC by Place of Delivery

The differentials in use of PNC by place of birth were substantial; the use being much higher in the case of facility births compared to home births. Not surprisingly, the use of postnatal care was limited for mothers who gave birth at home (Figure 1). The findings based on concentration indices suggest higher socio-economic inequalities in selected components of postnatal care in case of home births when compared to facility births (Table 3). For example, the concentration index for a newborn check-up within 24 hours of births was much higher in case of mothers who had birth at home compared with mothers who had birth at health facility suggesting higher socio-economic inequalities in this aspect of PNC relating to home births in contrast with births at a facility. Comparing home births with facility births, the inequalities in receipt of two or more check-ups for the baby before 10 days of life were found to be more pronounced in case of home births. An interesting finding emerges when we examine the concentration indices for choice of facility for the newborn’s check-up. The socio-economic inequalities were much higher in choice of facility for PNC check-up of newborn in the case of facility births as compared with births at home.

**Table 6:** Adjusted probabilities of postnatal care use by maternal complications, India, 2007–08.

Covariate & Category	*Caesarean birth*					*Birth with maternal complications*				
	Mother received PNC check-upWithin48 hoursof birth^1^	Newborn received PNCcheck-up within24 hoursof birth^1^	Baby received2 or more PNC check-ups within 10 days^2^	Baby receivedPNCcheck-upin governmentfacility^1^	Baby received PNC check-up in private facility^1^	Mother received PNC check-upWithin48 hoursof birth^1^	Baby receivedPNC check-upwithin24 hoursof birth^1^	Baby received2 or morePNCcheck-upswithin10 days^1^	Baby receivedPNC check-upin governmentfacility^1^	Baby received PNC check-up in private facility^1^
**Wealth index**										
Poorest	0.58	0.58	0.71	0.46	0.25	0.21	0.22	0.37	0.40	0.11
Poorer	0.78	0.77	0.71	0.45	0.33	0.30	0.30	0.42	0.44	0.13
Middle	0.86	0.83	0.77	0.45	0.36	0.42	0.43	0.52	0.47	0.18
Richer	0.90	0.90	0.84	0.39	0.46	0.57	0.59	0.61	0.45	0.26
Richest	0.95	0.95	0.87	0.25	0.64	0.78	0.80	0.74	0.33	0.47
***Richest-Poorest***	0.37	0.37	0.16	-0.21	0.39	0.57	0.58	0.37	-0.07	0.36

Note: The means were adjusted for mother’s education, place of residence, region of residence, health insurance coverage, birth order, mother received any ANC, and sex of the child. 1 - Wald test significant at p<0.001.

**Figure 3:**
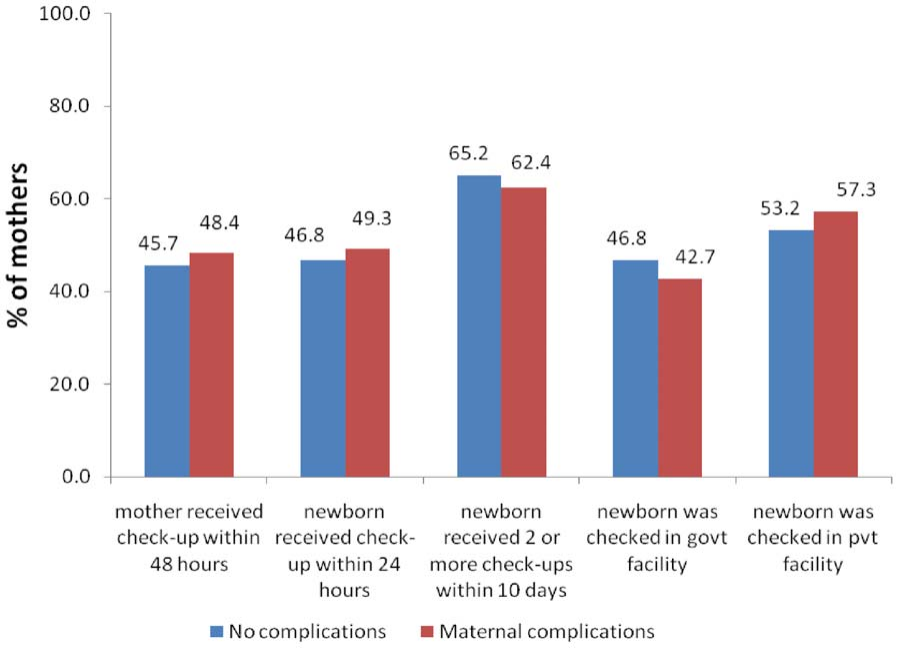
Differentials in postnatal care by experience of maternal complications.

The adjusted probabilities of PNC use, as derived from logistic regression model are presented in Table 4. Apart from the fact that use of PNC was distinctly lower among home births as against facility births, the poor-rich difference was maintained at almost similar levels. Across all components of PNC and the entire spectrum of wealth score there remained an advantage for use of PNC in case of facility births. The two components i.e. check-up at government facility and private facility depict a pattern conforming to the preference of the rich for private facility. Indeed the socio-economic inequalities in the use of PNC remained high even after adjusting for important socio-economic, demographic and residence related variables. Overall, the predicted probabilities suggest significantly higher use by the rich compared to the poor irrespective of the place of birth of newborn. However, the gap between the richest and poorest was found to be higher in case of facility births compared to home births.

### Inequality in PNC by Maternal Complications

Literature suggests that cesarean births and births with maternal complications are considered to be at high risk during the first few weeks of life. Though low birth weight babies and babies of small size are also at high risk, but they were not included in the analysis due to the unavailability of such information in the DLHS – 3 dataset. Therefore, the analysis focusses only on use of PNC among those who had a cesarean birth and those with maternal complications.

Figure 2 presents the use of PNC for cesarean deliveries. For comparison we have contrasted the same against the use of PNC for normal deliveries. Findings clearly suggest higher use of PNC for those who had cesarean deliveries compared to those with normal deliveries. Check-ups for mother and baby within 24 hours of birth were almost universal in the case of cesarean births. On the contrary, such examinations were limited in the case of normal births. However, even in the case of cesarean deliveries not all babies received two-or more check-ups within first 10 days of life. This percentage was even lower in the case of normal deliveries. Another observation relates to babies born from cesarean sections being more likely to get a check-up at a private facility as opposed to normal births examined at government facilites.

Estimated concentration indices suggest considerable socio-economic inequalities in the use of PNC even for mothers who had cesarean births. This is an important finding given the fact that cesarean sections occur in health facilities and women remain in the facility for a prolonged period of time after the procedure. This should in theory facilitate closer interaction between the mother and the health provider and results in better opportunities to receive appropriate PNC and related counselling services. Though the levels of inequalities were low in three out of five components of PNC, the inequalities were particularly pronounced in choice of place for PNC. The poor were more likely than the rich to access care in a government facility, whereas, the rich tend to utilize private facilities for PNC (Table 5).

The adjusted probabilities of accessing PNC by wealth quintile are presented in Table 6. The results adjusted for socio-economic, demographic and residence related variables suggest that rich were much more likely than the poor to utilize PNC and that were also more likely to utilize private facility for availing such care.

Unlike the case of cesarean births, no consistent association was found between maternal complications at the time of birth and subsequent use of PNC (Figure 3). Some of the components had greater use among mothers who did not have complications during birth, while the others were more likely to be used by those mothers with reported complications. Births with maternal complications were no different from normal births in receiving the recommended set of PNC. This finding contradicts our belief that mothers with complications during birth should be using PNC more often than those without complications.

However, we observe socio-economic inequalities in use of PNC even among mothers who experienced complications during birth. The concentration indices for the first three components were 0.074, 0.236 and 0.109, suggesting significant socio-economic inequality in the use of these three components (Table 5). Even in case of births with maternal complications, the poor were more likely than the rich to utilize government facilities whereas the rich were more likely to utilize private facilities. The logistic regression results confirm the descriptive findings that the rich availed PNC more than the poor. In some components the differences were of the order of 0.57–0.58 (Table 6).

## Discussion

This research is the first of its kind to present national level analyses of PNC in India. Such an attempt is motivated by the premise of asserting whether inequalities in utilisation of antenatal and childbirth care persist further in PNC as well. The assessment of such inequalities is made across various risk indicators such as cesarean section and births with maternal complications. The findings demonstrate clear evidence that PNC use has been unacceptably low and inequitable in India. The levels of PNC are relatively much lower than those of antenatal and childbirth care. Coverage of PNC is extremely unequal with the richest mothers having better access than their poorer counterparts.

There is further evidence that effective use of pregnancy and childbirth care in health facilities lead to better PNC [30]. Yet, there are wide socio-economic inequalities in those accessing facility based care. Overall, the coverage of essential PNC is limited especially for mothers from marginalised and economically disadvantaged households. The findings also suggest high inequalities in the type of PNC for births that took place at home compared to facility births – with postnatal check-ups within 24 hours and two or more PNC check-ups within the first 10 days of birth being particularly unequal. The risks associated with particularly home births could accumulate due to poor follow-up after the birth. Contrary to our expectations, the use of PNC was also unequitable in the case of facility births, which raises important questions about the functioning and quality of maternal health care services in India. Existing evidence highlight access and cost factors determining antenatal and childbirth care in India including poor transportation, indirect costs and out-of-pocket expenditure associated with care and more importantly poor quality of maternal health care services [31]–[37]. The present findings are indicative of the fact that part of the non-use and inequalities in maternal health care can be explained by the inherent drawbacks in health systems which are inclined to treat clients based on their socio-economic position. There is, therefore, an urgent need to revamp the maternal health care system in India by one that is efficient in service delivery without any form of discrimination and is also sensitive to the needs of clients.

Interestingly, the findings indicate socio-economic inequality in postnatal care use even in the case of cesarean births. This is quite surprising given the fact that women are expected to stay longer in facilities after a cesarean section. We did not find any consistent relationship between mothers reporting complications at childbirth and PNC use – despite the rich-poor differences. Women who had complications at birth usually need systematic follow-up to monitor and manage health risks for both the mother and her child [14]. The present analyses show that even among this group of women, rich women were particularly more likely than the poor women to seek care during the postnatal period.

The finding that poor and the marginalized use PNC more from government health facilities is important. Although government facilities minimize the economic burden of healthcare for the poor, the services available are often of sub-standard quality. Postnatal interventions should address the quality of care in government facilities to reduce the inequalities between the rich and the poor.

The potential limitations of the study should be noted. It has to be noted that maternal complications are self-reported by mothers based on their experiences and perceptions, and hence, the observed relationship between maternal complications and PNC should be interpreted with caution. The present study could not examine the quality of PNC services offered in government/private facilities. Also, there is a lack of information in the DLHS data on whether mothers who had cesarean births had PNC before or after discharge from the facility. Ideally, the questions on postnatal care use in case of cesarean births must refer to PNC use after discharge from the facility. This is because women stay in the facilities for longer periods after cesarean births. Finally, this research could not externally validate the survey responses, although the trends seen in various rounds of DLHS ensure consistency across time. On the other hand, the survey format is standard and regularly used in the various rounds of the DLHS. Survey teams receive formal training and essential quality control measures are in place.

The findings hold implications for the policies and programmes aimed at improving maternal health in India. One of the important interventions for improving maternal and child health under NRHM has been the conditional cash transfer scheme more widely known as the *Janani Suraksha Yojana* (JSY) [38]. Under this scheme, pregnant women are offered with cash incentives if they opt to giving a birth in a health facility, public or a government designated private hospital. However, there are no concurrent schemes in place to promote postnatal care.

Whilst it is important to promote institutional births especially in India where population level risks of maternal and child mortality are very high, essential postnatal care can have additional and substantial benefits in enhancing maternal and child health outcomes and wellbeing at later life [37]. Moreover, essential postnatal care could help in reducing postpartum depression among new mothers – a topic that has not received adequate attention in the country [39]. India urgently needs a comprehensive maternal health package that addresses the spectrum of maternal and extended newborn care – both components envisaged critical in achieving the targets 4, 5a and 5b of the UN Millennium Development Goals [40]. Targeted policy interventions such as health promotion and knowledge campaigns are needed to strengthen the postnatal care component in the maternal health care system.

In order to have a better understanding of the changes in maternal and child health inequalities there has to be regular monitoring and analysis of health indicators – such as postnatal care - and associated inequalities. Further analyses stratified by geographic region of residence and states will offer better policy insights of the heterogeneity of PNC across diverse social and cultural settings. The present study could not incorporate the geographical effects since the analysis explicitly focussed to establish evidence at the national level. Given the socio-economic and geographic diversity in India, large-scale surveys like DLHS should collect more information on postnatal care to allow detailed analysis of postnatal care at the state and regional levels.
